# Acute bilirubin encephalopathy and its associated risk factors in a tertiary care hospital, Pakistan

**DOI:** 10.12669/pjms.36.6.2222

**Published:** 2020

**Authors:** Munir Ahmad, Abdur Rehman, Mudasser Adnan, Muhammad Khalil Surani

**Affiliations:** 1 Dr. Munir Ahmad (FCPS Pediatric Medicine), Medical Officer Pediatric Medicine, The Children’s Hospital & The Institute of Child Health, Multan, Pakistan; 2 Dr. Abdur Rehman (FCPS Pediatric Medicine, FCPS Neonatology), Assistant Professor, The Children’s Hospital & The Institute of Child Health, Multan, Pakistan; 3 Dr. Mudasser Adnan (FCPS Pediatric Medicine), Senior Registrar Pediatric Medicine, The Children’s Hospital & The Institute of Child Health, Multan, Pakistan; 4 Dr. Muhammad Khalil Surani (FCPS Pediatric Medicine), Senior Registrar Pediatric Medicine, The Children’s Hospital & The Institute of Child Health, Multan, Pakistan

**Keywords:** Acute bilirubin encephalopathy, Hyperbilirubinemia, Pre-term birth, Mortality

## Abstract

**Objective:**

To determine the incidence of acute bilirubin encephalopathy (ABE) and its risk factors in neonates presenting with hyperbilirubinemia in a tertiary care children hospital.

**Methods:**

This descriptive observational study was conducted from June 2018 to June 2019. A total of 300 infants who were admitted in neonatal ICU with diagnosis of hyperbilirubinemia in The Children’s Hospital & The Institute of Child Health, Multan, Pakistan were included in this period. Incidence of ABE was noted. ABE was divided into two categories on the basis of severity of symptoms; mild ABE and moderate to severe ABE. Total serum bilirubin (TSB) in all neonates was measured in all patients in hospital laboratory using colorimetric method. ABO incompatibility and Rh factor incompatibility was also noted for each neonate.

**Results:**

Out of 300 neonates who presented with hyperbilirubinemia, ABE was diagnosed in only 42 (14.0%) neonates (mild ABE in 17 (5.7%) and moderate in 25 (8.3%). Out of 42 neonates of ABE, total serum bilirubin levels were 20-29.9 mg/dL in 24 (40.5%) neonates, and >30 mg/dL in 18 (42.8%) neonates. Pre-term birth was a significant risk factor of ABE; 23.8% in ABE and 10.70% in non-ABE (p-value 0.01). During treatment, 02 (4.76%) neonates expired due to ABE.

**Conclusion:**

In present study, ABE was diagnosed in 14.0% neonates who presented with hyperbilirubinemia. We found pre-term delivery as a significant risk factor of ABE.

## INTRODUCTION

Hyperbilirubinemia is very common in early neonatal period. Bilirubin levels rise for first few days of life and then return to normal up to the end of first week of life.^1^ In few neonates’ bilirubin levels rise to such a high level that these crosses the blood brain barrier and results in acute bilirubin encephalopathy (ABE).^2^ ABE is a group of clinical/pathologic abnormalities that results from bilirubin toxicity in central nervous system (CNS). According to American academy of pediatrics (APP) ABE is an acute manifestation seen in the first week of life after birth.^3^ ABE is one of the highly prevalent diseases of neonates and is a major cause of hearing impairment, developmental delay and cerebral palsy especially in developing countries.^4^,^5^ ABE is associated with 5% to 14% higher risk of deaths and 2 to 3 folds’ higher risk of neurological defects in neonates who survive from ABE.^6^ ABE has three phases, disease is reversible in first phase, but there is 2^nd^ and 3^rd^ phase is associated with higher risk of kernicterus (due to chronic bilirubin encephalopathy (CBE)).^7^

Risk factors of ABE varies from region to region, such as male gender, rhesus iso-immunisation and gestational age 35-38 years are common factors in Europe.^8^ A systemic review conducted in developing countries including Pakistan reported sepsis, G6PD deficiency, rhesus iso-immunisation, and low birth weight and small gestational age as common factors leading to ABE.^9^

The exact incidence of ABE is not available for developing countries because of delays in diagnosis, flaws in reporting, and absence of database management systems for collection of disease information.^5^ A study from Baghdad reported ABE in 1749 neonates per 100,000 live births.^10^ In Pakistan, data regarding ABE is very scarce. So we aimed to determine the incidence of ABE and its risk factors in neonates presenting with hyperbilirubinemia in a tertiary care child hospital.

## METHODS

This descriptive observational study was conducted from June 2018 to June 2019. A total of 300 infants who were admitted in neonatal ICU with diagnosis of hyperbilirubinemia in The Children’s Hospital & The Institute of Child Health, Multan were studied in this period. Approval from ethical committee of the hospital was taken (EC No: 62, Dated: 20-01-2020).

Incidence of ABE was noted. ABE was divided into two categories on the basis of severity; ***mild ABE;*** presence of poor feeding, lethargy, hypo/hyper-tonia, or high pitch crying voice. ***Moderate to Severe ABE;*** presence of retro-collis, rigidity or flaccidity, opisthotonus, paralysis of upward gaze, apnea, or seizures. Mortality due to ABE was also noted.

Total serum bilirubin (TSB) in all neonates was measured in all patients in hospital laboratory using colorimetric method. ABO incompatibility and Rh factor incompatibility was also done for each neonate diagnosed with encephalopathy. ABE due to hemolytic anemia was diagnosed if TSB level ≥20 mg/dl and hematocrit (HCT) ≤35% in neonate.

After confirming the diagnosis of ABE, the ABE infants were categorized into two categories depending upon the TSB levels; TSB 20-29.9 mg/dL were labelled as at threshold of intervention, while those having ≥30 mg/dL, at high risk of development of kernicterus. Risk factors of ABE such as preterm delivery, place of delivery, ABO/Rh incompatibility were noted. For data analysis we used SPSS v23 software. Risk factors of ABE, in ABE and non-ABE neonates were compared using chi-square test. P-value ≤0.05 was taken as significant.

## RESULTS

Out of 300 neonates who presented with hyperbilirubinemia, ABE was diagnosed in only 42 (14.0%) neonates (mild ABE in 17 (5.7%) and moderate to severe in 25 (8.3%). Out of 42 neonates of ABE, TSB levels were 20-29.9 mg/dL in 21 (50.0%) neonates, >30 mg/dL in 04 (9.5%) neonates, while <20 in 17 (40.5%) neonates. During treatment, 02 (4.76%) neonates expired due to ABE (Table-I).

**Table-I:** ABE Major Characteristics and Outcomes.

Total ABE	42 (14.0%)
***ABE Severity***	
Mild ABE	17 (5.7%)
Moderate to severe ABE	25 (8.3%)
***Total Serum Bilirubin levels in ABE infants.***	
TSB <20 mg/dL	17 (40.5%)
TSB 20-29.9 mg/dL	21 (50.0%)
TSB >30 mg/dL	04 (9.5%)
Mortality	2 (4.76%)

Regarding risk factors of ABE, there were 10 (23.8%) preterm neonates in ABE neonates, and 26 (10.70%) preterm neonates in non-ABE group (p-value 0.01). There was no significant difference in place of birth and birth weight between the groups. There was no significant difference in ABO and Rh incompatibility between the ABE and non-ABE neonates. (Table-II).

**Table-II:** Risk Factors Associated with ABE.

	ABE (N=42)	Non-ABE (n=258)	p-value
Pre-term Birth	10 (23.8%)	26 (10.7%)	0.01
***Place of Birth***			
Hospital	13 (30.9%)	115 (44.6%)	0.09
Clinic	29 (69.1%)	143 (55.4%)
***Birth Weight***			
<1500 grams	02 (4.7%)	08 (3.10%)	0.41
1500-2499 grams	14 (33.3%)	113 (43.8%)	
>2500 grams	26 (62.0%)	137 (53.1%)
***Other Hematologic Causes of Hyperbilirubinemia***			
ABO Incompatibility	12 (28.6%)	80 (31.0%)	0.75
Rh Factor Incompatibility	1 (2.4%)	14 (17.5%)	0.40

## DISCUSSION

ABE is still a problem in developing countries. The American National quality forum has defined death or disability (kernicterus) as a very serious event that results from failure of early diagnosis or failure to treat hyperbilirubinemia and stressed that kernicterus should become a “never event” in the world.^11^ However, in poor countries due to the availability of limited resources and proper neonatal facilities has posed a major challenge for making Kernicterus a never event. Many of the neonates of hyperbilirubinemia in developing nations present late in hospital after development of symptoms of ABE. Many studies have reported higher incidence of ABE among neonates who are born in out-hospital facilities.^12^-^14^

Some studies have reported a direct association between TSB and risk of ABE. Iskander et al. and Kuzniewicz et al. reported that the risk of ABE is lower in neonates having TSB <30 mg/dL.^15^,^16^ While a study by Ogunlesi et al. reported ABE in 38.26% neonates having TBS <20 mg/dl, some of these neonates were suffering from sepsis or were pre-term.^17^ In present study there were 40.5% neonates having TSB <20 mg/dL, there were only 9.5% neonates having TSB >30 mg/dL.

Studies have also reported that place of birth also effects the risk of ABE.In out-of-hospital birth places there is a poor knowledge regarding hyperbilirubinemia and its treatment options.^18^,^19^ A study conducted by Diala et al. reported a strong association between the in home delivery and frequency of ABE.^18^ In our study, there was no case of home birth and we did not find any significant difference in incidence of ABE in clinic birth and in-hospital birth neonates. A study from Pakistan by Korejo et al. reported home delivery and late presentation as risk factors of kernicterus in hyperbilirubinemia neonates.^20^

In present study, we found pre-term delivery as a significant risk factors of ABE. We found preterm birth in 23.8% neonates in ABE neonates and in 10.70% neonates in non-ABE neonates. A study conducted in Myanmar by Arnolda et al. also reported opposite results. In their study pre-term birth rate was 8.3-9.4% in ABE neonates versus 16.1-30.3% in non-ABE neonates.^21^ A recent review by Bhutani et al., concluded that pre-term neonates are at increased risk of progression to ABE and more likely to develop long term neurological complications either due to ABE or due to overtreatment of phototherapy or exchange transfusion.^22^

We did not find any significant difference in ABO incompatibility and Rh incompatibility. Arnolda et al. also reported similar findings.^20^ Whoever a study by Bhutani et al. reported that Rh incompatibility is responsible for nearly 1/3^rd^ all incidences of ABE.^5^

### Significance of study

Our study is one of the few studies conducted in Pakistan that have determined the risk factors of development of ABE in neonatal hyperbilirubinemia. Identification of risk factors can help in determining the high risk hyperbilirubinemia neonates in whom ABE can occur.

### Limitations of the study

It’s a single center study and we only included the neonates who presented with hyperbilirubinemia. Moreover, our hospital is situated in a dense urban population area. So this study cannot determine the true incidence of ABE. Because most of population of Pakistan nearly 70% belongs to rural areas and many births occur in basic health facilities or sometimes at home. So there is a need to conduct multi-center study to determine the exact incidence of ABE in our population and to determine the risk factors associated with ABE.

## CONCLUSION

In present study, ABE was diagnosed in 14.0% neonates who presented with hyperbilirubinemia. We found pre-term delivery as a significant risk factor of ABE.

### Authors Contribution:

**MA:** Conceived, designed the research methodology and is responsible for integrity of study.

**AR:** Supervised the research project, did editing.

**MA & MKS:** helped in data collection and compilation.
